# Measurement of Aerosol Particles from Vibrated Lab Coats

**DOI:** 10.3390/toxics12080565

**Published:** 2024-08-02

**Authors:** Byung Uk Lee

**Affiliations:** Bioaerosol Laboratory, Konkuk University, 120 Neungdong-ro, Gwangjin-gu, Seoul 05029, Republic of Korea; leebu@konkuk.ac.kr; Tel.: +82-2-450-4091

**Keywords:** lab coat, vibration, aerosol, clothing, emission, shirt, suit

## Abstract

A study was conducted to measure aerosol particles emitted from laboratory coats (lab coats) under vibration, comparing them with a suit and a shirt. This study focused on particles ranging from 0.3 μm to >10 μm. Experimental results showed that lab coat vibration increased particles >5 μm while reducing submicron particles. The lab coat (old and used) exhibited greater particle concentration variations under vibration compared to those using the new lab coat or the shirt. Contrastingly, the suit under vibration did not significantly affect particle concentrations. These findings highlight the impact of lab coat vibration on aerosol particle concentrations in the surrounding air, which is important for work environments.

## 1. Introduction

Aerosol particles have been identified as a causative factor of public health issues for a prolonged period [1,2]. These particles, originating from various sources, have the potential to induce diseases upon entering human respiratory tracts or adhering to compromised skin [3,4,5,6,7,8,9]. Airborne ultrafine particles were found in the cells of rats that were exposed to them [3], and specific nanomaterials have been known to be toxic to the respiratory system [4]. Aerosol particles have been considered carriers of respiratory pathogens [5], and some atmospheric particulate matter has been suspected to have selective potential neurotoxicity [6]. Wood tar aerosols were reported to affect the lung cells of mice [7], and it was reported that air pollutants, including aerosol particles, showed correlations with respiratory health [8,9]. Among the diverse array of particle sources, clothing emerges as a particularly influential factor in the vicinity of humans, as it frequently comes into close contact with human life and readily attracts environmental particles. Specifically, in environments such as hospitals or healthcare facilities, airborne viral particles may adhere to the clothing of healthcare workers and patients. If these particles dislodge from the clothing under certain conditions, they can contribute to the transmission of viral diseases to other individuals who come into contact with them [10,11,12,13]. Furthermore, airborne hazardous particles, including toxins, may adhere to the clothing of workers exposed to them in occupational settings such as chemical factories. If these particles detach from the workers’ clothing, they pose a potential threat to public health [14,15]. Lab coats, commonly worn in healthcare facilities and various industrial settings, serve as representative examples of clothing types that warrant investigation as potential sources of aerosol particles. In this study, the total aerosol particles detached from lab coats were measured when they were in a vibration state, and the size distributions of the detached particles were analyzed. A suit and a shirt were also tested for comparison with lab coats.

## 2. Materials and Methods

Two types of lab coats were tested: (1) used lab coat (Lab Coat A) and (2) new lab coat (Lab Coat B). Additionally, two other types of clothing, (3) a suit and (4) a shirt (T-shirt), were tested for comparison with the lab coats. Overall, four types of clothing were evaluated (dimensions and other material information are provided in Table 1).

In the first experiment, a used lab coat (Lab Coat A) was vibrated at a frequency of 1 Hz for 10 s with 5 cm amplitude, and the variation in aerosol particle concentrations was studied. Experimental conditions were selected to replicate a practical lifetime of vibrations associated with running or active walking. The concentrations of aerosol particles were measured before and after the vibration activity using a laser optical particle counter (Portable particle counter, Model 3905, KANOMAX, Andover Township, NJ, USA) to measure both concentration and size distributions [16]. The measured aerosol particles ranged from 0.3 μm to ≥ 10 μm based on their optical sizes. The laser optical particle counter has six channels for particle sizes: 0.3 μm (0.3 μm ≤ dp < 0.5 μm), 0.5 μm (0.5 μm ≤ dp < 1 μm), 1 μm (1 μm ≤ dp < 3 μm), 3 μm (3 μm ≤ dp < 5 μm), 5 μm (5 μm ≤ dp < 10 μm), and 10 μm (10 μm ≤ dp). The laser optical particle counter detected the laser scattering signals induced by passing aerosol particles in laser radiation light, and the signals were translated into the data for particle concentrations and particle sizes with calibration processes [16].

For each size section of aerosol particles, the initial concentration before the vibration experiments and the final concentrations after the vibration experiments were compared. The experiments were conducted inside an artificially manufactured confined experimental device, a rectangular wooden box (0.3 m × 0.3 m × 1.5 m) prepared specifically for this study. The experimental device was placed inside the research building of Konkuk University, Seoul. The vibration experiment was performed using a steering rod connected to the clothing to be tested, along with the examination of time. At least three replication experiments were conducted under each condition. For sampling and measuring the levels of particles in the air inside the experimental device, a measurement tool was operated within the device, and no external wind occurred.

In the second, third, and fourth experiments, other types of clothing, such as a new lab coat (Lab Coat B), a suit, and a shirt (T-shirt), were tested for their vibration effects following the same procedure as that used for the used lab coat (Lab Coat A) experiment, respectively.

## 3. Results

### 3.1. Experimental Results with Lab Coats (Used Lab Coat and New Lab Coat)

**Figure 1:**
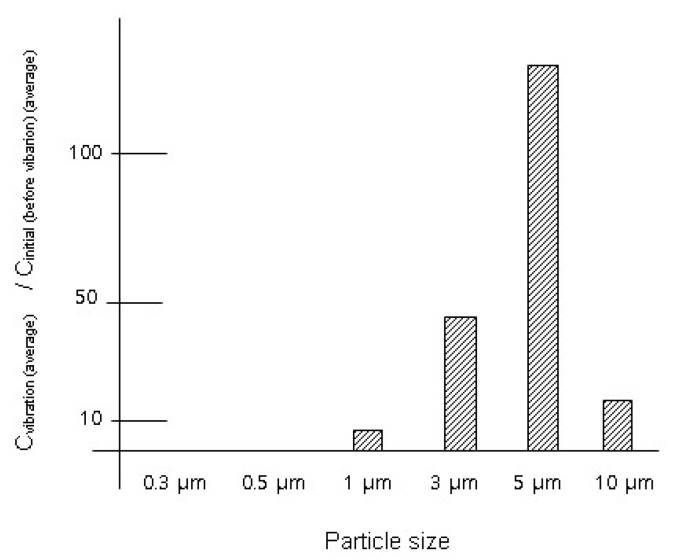
Variation ratio (vibration of Lab Coat A): C_vibration (average)_/C_initial(average)_.

Table 2 and Figure 1 present the experimental results regarding the effect of a used lab coat (Lab Coat A). Concentrations of particles larger than 1 μm (≥1 μm) increased significantly when Lab Coat A was subjected to 1 Hz vibrations for 10 s. Specifically, particles sized between 5 μm and 10 μm (5 μm ≤ dp < 10 μm) exhibited the most notable increase among various particle sizes. The concentration of these large particles varied from 2.93 × 10^4^ ± 1.63 × 10^4^ particles/m^3^ to 3.80 × 10^6^ ± 9.01 × 10^5^ particles/m^3^, representing a 130-fold increase compared to the initial concentration before the vibration experiment, with statistical significance (*t*-test *p*-value < 0.05). However, concentrations of particles sized between 0.3 μm and 1 μm (0.3 μm ≤ dp < 1 μm) decreased significantly after the vibration of Lab Coat A. The concentration of these small particles was less than 0.1% of the initial concentration.

In the case of the new lab coat (Lab Coat B), concentrations of particles larger than 1 μm (≥1 μm) increased, similar to the results observed with the used lab coat (Lab Coat A) in the experiment subjected to 1 Hz vibrations for 10 s, as illustrated in Table 3. However, in this instance, concentrations of particles with sizes larger than 10 μm (dp ≥ 10 μm) exhibited the most significant increase among particles of various sizes. The concentration of these large particles varied from 2.59 × 10^3^ ± 8.89 × 10^2^ particles/m^3^ to 1.11 × 10^5^ ± 1.41 × 10^4^ particles/m^3^, indicating a 43-fold increase compared to the initial concentration, with statistical significance (*t*-test *p*-value < 0.05) attributed to vibration stimuli.

### 3.2. Experimental Results with the Suit

**Figure 2:**
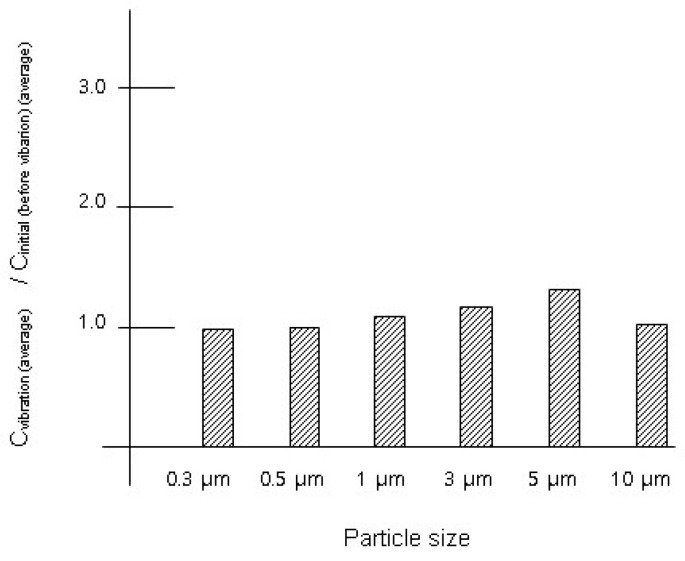
Variation ratio (vibration of suit): C_vibration (average)_/C_initial (average)_.

The suit underwent testing to compare their effects with those of lab coats. The experimental results are presented in Table 4 and Figure 2. Vibrations from the suit led to minimal changes in aerosol particle concentrations. The concentration range of measured particles fluctuated by less than 31% (<31%) in response to 1 Hz vibrations lasting 10 s. Concentrations of aerosol particles sized between 5 μm and 10 μm increased from 8.56 × 10^4^ ± 8.97 × 10^3^ particles/m^3^ to 1.12 × 10^5^ ± 1.08 × 10^4^, indicating a 31% increase compared to the initial concentration, although this increase was not statistically significant (*t*-test *p*-value > 0.05). However, post-vibration concentrations of aerosol particles smaller than 5 μm (<5 μm) remained comparable to initial levels.

### 3.3. Experimental Results with the Shirt

The shirt (T-shirt) was also tested to compare its effects with those of lab coats. Table 5 presents the experimental results for the shirt. Aerosol particle concentrations changed due to shirt vibrations. Concentrations of particles larger than 1 μm (≥1 μm) increased when the shirt was subjected to 1 Hz vibrations for 10 s. Specifically, particles sized between 5 μm and 10 μm (5 μm ≤ dp < 10 μm) exhibited the most notable increase among various particle sizes. Concentrations of aerosol particles sized between 5 μm and 10 μm (5 μm ≤ dp < 10 μm) increased from 8.06 × 10^4^ ± 3.24 × 10^3^ particles/m^3^ to 1.84 × 10^6^ ± 4.37 × 10^5^ particles/m^3^, indicating a 23-fold increase compared to the initial concentration. However, concentrations of particles sized between 0.3 μm and 0.5 μm (0.3 μm ≤ dp < 0.5 μm) decreased significantly after shirt vibrations, dropping from 2.96 × 10^7^ ± 3.83 × 10^5^ particles/m^3^ to 2.86 × 10^6^ ± 2.20 × 10^6^ particles/m^3^, less than 10% of the initial concentration. Overall, variations in particle concentrations due to shirt vibrations were similar to those observed with lab coats.

## 4. Discussion

Vibrations from the used lab coat (designated as Lab Coat A) induced variations in aerosol particle size distributions. The concentration of particles larger than 5 μm significantly increased, while the concentration of submicron particles (<1 μm) markedly decreased due to the vibration. This phenomenon can be elucidated by several hypothetical mechanisms. Firstly, the vibration effect of the used lab coat within the experimental air environment led to the coagulation and transformation of submicron particles into larger particles. The vibrational activity of the lab coat may induce the formation of electrical charges on submicron particles due to triboelectrification (static electrification) [2], thereby accelerating their coagulation into larger particles [1,2]. Secondly, particles larger than 5 μm could have been emitted or detached from the used lab coat (Lab Coat A) during the vibration. These detached particles may have contributed to an increase in the concentration of larger aerosol particles. 

These two explanations can be expressed using the following differential equations for the concentration of the aerosol particles: Equation (1) is a differential equation of specific-sized aerosol particle concentrations, “*n_k_*”, in the used lab coat (Lab Coat A) experiment. In this differential equation, “*t*”, “*V*”, and “*D*” refer to time, air velocity, and a diffusion coefficient, respectively [1,2,16,17]. Mathematical operators in vector calculus are used in this differential equation [18].

(1) $$\frac{\partial n_{k}}{\partial t}+\nabla \cdot n_{k}\vec{v}={\left(\nabla \cdot D\nabla n_{k}\right)}_{diffusion}+{\left[\frac{\partial n_{k}}{\partial t}\right]}_{generation}-{\left[\frac{\partial n_{k}}{\partial t}\right]}_{coagulation(out)}+{\left[\frac{\partial n_{k}}{\partial t}\right]}_{coagulation(in)}$$

For particles of sizes from 0.3 μm to 1 μm, coagulations “$-{\left[\frac{\partial n_{k}}{\partial t}\right]}_{coagulation(out)}$” due to vibration effects transformed these particles (decrease in concentration) to particles of sizes larger than 1 μm (>1 μm) (increase in concentration). For particles of sizes >5 μm, both external inputs of particles detached from the used lab coat (Lab Coat A) vibrations, which was expressed as “${\left[\frac{\partial n_{k}}{\partial t}\right]}_{generation}$” in Equation (1), and coagulation results generated from submicron and small particles, which was expressed as “${\left[\frac{\partial n_{k}}{\partial t}\right]}_{coagulation(in)}$” in Equation (1), increased the concentration of these particles.

The vibration of the suit caused little variation in the aerosol particle size distributions, as shown in Table 4. This phenomenon can be understood using differential Equation (1) for the concentration of aerosol particles. For the case of the suit, coagulations for submicron particles were weak, “$-{\left[\frac{\partial n_{k}}{\partial t}\right]}_{coagulation(out)}$”; therefore, no significant transformation of these particles (decrease in concentration) to larger particles was observed. The vibration of the suit, which mostly consists of vinyl-type polyester, may not cause significant electric charges on the aerosol particles; therefore, coagulation did not occur actively. For particles of sizes > 5 μm, the artificial particle input due to vibration of the suits, which was expressed as “${\left[\frac{\partial n_{k}}{\partial t}\right]}_{generation}$” in Equation (1), was low, and the coagulation input effects, which were connected to the coagulation effects of submicron particles, were not significant; therefore, the concentration of these larger (>5 μm) aerosol particles did not vary significantly. It can be inferred that the suit did not emit aerosol particles into the surrounding air environments when subjected to vibrations.

The experimental results for the new lab coat (Lab Coat B) and the shirt can be understood as results in the middle of cases between the used lab coat (Lab Coat A) and the suit. 

The vibration condition tested is one example of the various vibrations associated with clothing. Specifically, running or active walking stimuli with a frequency of 1 Hz were simulated in this study. In the future, various clothing-specific stimuli need to be tested to understand this phenomenon comprehensively. In addition, the electrical and chemical properties of emitted particles can be analyzed for an extensive understanding of this phenomenon and the above hypotheses on the experimental results.

## 5. Conclusions

The vibration of lab coats has been observed to influence aerosol particle size distributions in surrounding environments. Vibrations from both used and new lab coats can significantly increase concentrations of particles >3 μm while simultaneously decreasing the concentration of submicron-sized aerosol particles. The main reasons for this phenomenon are estimated to be the emission of large particles from the vibrated clothing and the increased coagulation of submicron particles due to electric charges generated by the vibrating clothing. In contrast to lab coats, the vibration of the suit did not significantly affect aerosol particle concentrations in the surrounding environments. Further studies are required to elucidate the underlying causes of these results, including detailed investigations into the mechanisms of particle emissions and coagulation.

Furthermore, this study tested four types of clothing, which highlights a limitation in this study due to the limited number of clothing types examined. In addition, more experimental conditions can be considered in future research. Overall, it has been demonstrated that the vibration of lab coats in working environments has the potential to affect aerosol particle concentration conditions in surrounding air environments. Therefore, this factor should be taken into consideration when conducting any work that involves the vibration of lab coats.

## Figures and Tables

**Table 1:** Tested clothing.

	Lab Coat A	Lab Coat B	Suit	Shirt (T-Shirt)
Dimensions	Length: 110 cm Width: 45 cm	Length: 110 cm Width: 45 cm	Length: 85 cm Width: 45 cm	Length: 70 cm Width: 45 cm
Material	Polyester (50%) + Cotton (50%)	Polyester (65%) + Cotton (35%)	Polyester (100%)	Polyester (40%) + Cotton (60%)
Used/New	Used (~10 years)	New (0 years)	Used (~3 years)	Used (~3 years)

**Table 2:** Particle concentrations after vibration of Lab Coat A for 10 s.

	Particle Concentration (Initial Condition, C_initial_) Particles/m^3^	Particle Concentration (after Vibration of Lab Coat A for 10 s, C_vibration_) Particles/m^3^	Variation Ratio: C_vibration (average)_/C_initial (average)_
Particle size	Average ± standard deviation	Average ± standard deviation	Ratio with [*t*-test *p*-value]
0.3 μm	3.23 × 10^7^ ± 5.28 × 10^5^	1.80 × 10^4^ ± 3.12 × 10^4^	5.6 × 10^−4^ [4.9 × 10^−5^]
0.5 μm	3.40 × 10^6^ ±1.68 × 10^5^	0 × 10^4^ ± 0 × 10^4^	0 [4.1 × 10^−4^]
1 μm	7.15 × 10^5^ ± 2.01 × 10^4^	5.11 × 10^6^ ± 7.54 × 10^5^	7.1 [4.9 × 10^−3^]
3 μm	4.66 × 10^4^ ± 1.98 × 10^4^	2.11 × 10^6^ ± 5.88 × 10^5^	45 [0.014]
5 μm	2.93 × 10^4^ ± 1.63 × 10^4^	3.80 × 10^6^ ± 9.01 × 10^5^	130 [0.0096]
10 μm	6.83 × 10^3^ ± 3.49 × 10^3^	1.15 × 10^5^ ± 1.17 × 10^5^	17 [0.12]

**Table 3:** Particle concentrations after vibration of Lab Coat B for 10 s.

	Particle Concentration (Initial Condition, C_initial_) Particles/m^3^	Particle Concentration (after Vibration of Lab Coat B for 10 s, C_vibration_) Particles/m^3^	Variation Ratio: C_vibration (average)_/C_initial(average)_
Particle size	Average ± standard deviation	Average ± standard deviation	Ratio with [*t*-test *p*-value]
0.3 μm	3.09 × 10^7^ ± 3.76 × 10^5^	3.75 × 10^6^ ± 1.49 × 10^6^	0.12 [0.00065]
0.5 μm	3.50 × 10^6^ ± 1.50 × 10^5^	2.58 × 10^6^ ± 4.82 × 10^5^	0.74 [0.035]
1 μm	9.24 × 10^5^ ± 2.04 × 10^4^	6.95 × 10^6^ ± 6.15 × 10^5^	7.5 [0.0016]
3 μm	9.78 × 10^4^ ± 7.90 × 10^3^	1.57 × 10^6^ ± 2.07 × 10^5^	16 [0.0030]
5 μm	5.51 × 10^4^ ± 1.62 × 10^3^	1.45 × 10^6^ ± 3.06 × 10^5^	26 [0.0077]
10 μm	2.59 × 10^3^ ± 8.89 × 10^2^	1.11 × 10^5^ ± 1.41 × 10^4^	43 [0.0026]

**Table 4:** Particle concentrations after suit vibration for 10 s.

	Particle Concentration (Initial Condition, C_initial_) Particles/m^3^	Particle Concentration (after Vibration of Suit for 10 s, C_vibration_) Particles/m^3^	Variation Ratio: C_vibration (average)_ /C_initial (average)_
Particle size	Average ± standard deviation	Average ± standard deviation	Ratio with [*t*-test *p*-value]
0.3 μm	3.10 × 10^7^ ± 1.72 × 10^5^	3.03 × 10^7^ ± 3.26 × 10^5^	0.98 [0.043]
0.5 μm	3.34 × 10^6^ ± 4.47 × 10^4^	3.35 × 10^6^ ± 3.17 × 10^4^	1.00 [0.18]
1 μm	9.60 × 10^5^ ± 2.42 × 10^4^	1.05 × 10^6^ ± 1.85 × 10^4^	1.09 [0.032]
3 μm	1.21 × 10^5^ ± 8.84 × 10^3^	1.41 × 10^5^ ± 5.93 × 10^3^	1.17 [0.068]
5 μm	8.56 × 10^4^ ± 8.97 × 10^3^	1.12 × 10^5^ ± 1.08 × 10^4^	1.31 [0.072]
10 μm	1.22 × 10^4^ ± 2.86 × 10^3^	1.25 × 10^4^ ± 3.61 × 10^3^	1.02 [0.42]

**Table 5:** Particle concentrations after vibration of the shirt for 10 s.

	Particle Concentration (Initial Condition, C_initial_) Particles/m^3^	Particle Concentration (after Vibration of Shirt for 10 s, C_vibration_) Particles/m^3^	Variation Ratio: C_vibration (average)_ /C_initial (average)_
Particle size	Average ± standard deviation	Average ± standard deviation	Ratio with [*t*-test *p*-value]
0.3 μm	2.96 × 10^7^ ± 3.83 × 10^5^	2.86 × 10^6^ ± 2.20 × 10^6^	0.0970 [0.0015]
0.5 μm	3.50 × 10^6^ ± 8.59 × 10^4^	2.80 × 10^6^ ± 2.66 × 10^5^	0.799 [0.035]
1 μm	1.09 × 10^6^ ± 4.77 × 10^4^	8.53 × 10^6^ ± 1.93 × 10^6^	7.83 [0.011]
3 μm	1.24 × 10^5^ ± 7.78 × 10^3^	1.71 × 10^6^ ± 4.40 × 10^5^	13.8 [0.012]
5 μm	8.06 × 10^4^ ± 3.24 × 10^3^	1.84 × 10^6^ ± 4.37 × 10^5^	22.9 [0.0098]
10 μm	1.65 × 10^3^ ± 5.40 × 10^2^	2.41 × 10^4^ ± 2.31 × 10^4^	14.6 [0.12]

## Data Availability

The original contributions presented in this study are included in this Communication article, further questions can be directed to the author, B.U.L.

## References

[1] Friedlander S.K. (2000). Smoke, Dust, and Haze: Fundamentals of Aerosol Dynamics.

[2] Hinds W.C. (1999). Aerosol Technology: Properties, Behavior, and Measurement of Airborne Particles.

[3] Geiser M., Rothen-Rutishauser B., Kapp N., Schurch S., Kreyling W., Schulz H., Semmler M., Hof V.I., Heyder J., Gehr P. (2005). Ultrafine particles cross cellular membranes by nonphagocytic mechanisms in lungs and in cultured cells. Environ. Health Perspect..

[4] Kong C., Chen J., Li P., Wu Y., Zhang G., Sang B., Li R., Shi Y., Cui X., Zhou T. (2024). Respiratory Toxicology of Graphene-Based Nanomaterials: A Review. Toxics.

[5] Lee B.U. (2021). Why does the SARS-CoV-2 Delta VOC spread so rapidly? Universal conditions for the rapid spread of respiratory viruses, minimum viral loads for viral aerosol generation, effects of vaccination on viral aerosol generation, and viral aerosol clouds. Int. J. Environ. Res. Public Health.

[6] Liu F., Huang Y., Zhang F., Chen Q., Wu B., Rui W., Zheng J.C., Ding W. (2015). Macrophages treated with particulate matter PM2.5 induce selective neurotoxicity through glutaminase-mediated glutamate generation. J. Neurochem..

[7] Pardo M., Li C., He Q., Levin-Zaidman S., Tsoory M., Yu Q., Wang X., Rudich Y. (2020). Mechanisms of lung toxicity induced by biomass burning aerosols. Part. Fibre Toxicol..

[8] Wang D., Wang Y., Liu Q., Sun W., Wei L., Ye C., Zhu R. (2023). Association of Air Pollution with the Number of Common Respiratory Visits in Children in a Heavily Polluted Central City, China. Toxics.

[9] World Health Organization (WHO) (2016). Ambient Air Pollution: A Global Assessment of Exposure and Burden of Disease.

[10] Birgand G., Peiffer-Smadja N., Fournier S., Kerneis S., Lescure F.X., Lucet J.C. (2020). Assessment of Air Contamination by SARS-CoV-2 in Hospital Settings. JAMA Netw. Open.

[11] Gould S., Atkinson B., Onianwa O., Spencer A., Furneaux J., Grieves J., Taylor C., Milligan I., Bennett A., Fletcher T. (2022). Air and surface sampling for monkeypox virus in a UK hospital: An observational study. Lancet Microbe.

[12] Hernaez B., Muñoz-Gómez A., Sanchiz A., Orviz E., Valls-Carbo A., Sagastagoitia I., Ayerdi O., Martín R., Puerta T., Vera M. (2023). Monitoring monkeypox virus in saliva and air samples in Spain: A cross-sectional study. Lancet Microbe.

[13] Wang C.C., Prather K.A., Sznitman J., Jimenez J.L., Lakdawala S.S., Tufekci Z., Marr L.C. (2021). Airborne transmission of respiratory viruses. Science.

[14] Rayana T.B., Wild P., Debatisse A., Jouannique V., Sakthithasan K., Suarez G., Canu I.G. (2023). Job Exposure Matrix, a Solution for Retrospective Assessment of Particle Exposure in a Subway Network and Their Long-Term Effects. Toxics.

[15] Wu D., Li Q., Shang X., Liang Y., Ding X., Sun H., Li S., Wang S., Chen Y., Chen J. (2021). Commodity plastic burning as a source of inhaled toxic aerosols. J. Hazard. Mater..

[16] Lee B.U. (2020). Cryogenic Aerosol Generation: Airborne Mist Particles Surrounding Liquid Nitrogen. Int. J. Environ. Res. Public Health.

[17] Kreyszig E. (1999). Advanced Engineering Mathematics.

[18] Marsden J.E., Tromba A.J. (1988). Vector Calculus.

